# Cell penetrating peptides in preclinical and clinical cancer diagnosis and therapy

**DOI:** 10.18632/oncotarget.26442

**Published:** 2018-12-14

**Authors:** Prem Prakash Tripathi, Hamed Arami, Ivneet Banga, Jalaj Gupta, Sonu Gandhi

**Affiliations:** ^1^ CSIR-Indian Institute of Chemical Biology (CSIR-IICB), Kolkata, India; ^2^ IICB-Translational Research Unit of Excellence, Kolkata, India; ^3^ Molecular Imaging Program at Stanford (MIPS), The James H. Clark Center, Stanford University, Stanford, CA, USA; ^4^ Department of Radiology, Stanford University, School of Medicine, Stanford, CA, USA; ^5^ Department of Bioengineering, University of Texas, Arlington, TX, USA; ^6^ Institute for Tumor Biology and Experimental Therapy, Georg-Speyer-Haus, Frankfurt, Germany; ^7^ DBT-National Institute of Animal Biotechnology (DBT-NIAB), Hyderabad, India

**Keywords:** cell penetrating peptides, cancer targeting, therapy, diagnostic, imaging

## Abstract

Delivery of imaging reagents and drugs to tumors is essential for cancer diagnosis and therapy. In addition to therapeutic and diagnostic functionalities, peptides have potential benefits such as biocompatibility, ease to synthesize, smaller size, by-passing off-target side effects, and achieving the beneficial effects with lower-administered dosages. A particular type of peptide known as cell penetrating peptides (CPP) have been predominantly studied during last twenty years as they are not only capable to translocate themselves across membranes but also allow carrier drugs to translocate across plasma membrane, by different mechanisms depending on the CPP. This is of great potential importance in drug delivery systems, as the ability to pass across membranes is crucial to many drug delivery systems. In spite of significant progress in design and application of CPP, more investigations are required to further improve their delivery to tumors, with reduced side-effect and enhanced therapeutic efficacy. In this review, we emphasis on current advancements in preclinical and clinical trials based on using CPP for more efficient delivery of anti-cancer drugs and imaging reagents to cancer tissues and individual cells associated with them. We discuss the evolution of the CPPs-based strategies for targeted delivery, their current status and strengths, along with summarizing the role of CPPs in targeted drug delivery. We also discuss some recently reported diagnostic applications of engineered protease-responsive substrates and activable imaging complexes. We highlight the recent clinical trial data by providing a road map for better design of the CPPs for future preclinical and clinical applications.

## INTRODUCTION

Despite significant advances in anti-cancer therapies such as chemo-, radio-, and hormone therapies, surgical resection combined with chemo-radiotherapy remains the standard approach for fighting malignant cancers [1]. However, chemotherapy is not ideal because of its side-effects such as general damage to healthy cells and insufficient surgical resection usually results in cancer recurrence in various cases such as glioblastoma multiforme (GBM). During the last decade, antibodies and large protein-ligands were extensively studied in various targeted delivery-based clinical trials [2]. However, these biomolecules face many drawbacks such as poor delivery of the monoclonal antibodies (mAbs) to tumors due to their larger sizes that slow down the passive diffusion across the plasma membrane, thus affecting the uptake of these biomolecules at luminal side of tumor vessels [3, 4]. Moreover, non-specific uptake of mAbs by the liver also cause dose-dependent toxicity, thus they are non-ideal candidate for targeted delivery.

Peptides have emerged as alternatives for mAbs, because of their reduced non-specific toxic effects, rapid renal clearance, small size, high specificity, and efficient delivery to tumor [5]. They have higher tumor-specific binding compared to other targeting biomolecules, which makes them suitable candidates for targeted drug delivery without affecting surrounding normal cells. Various studies have documented the advantage of antibody mimicking small peptides in penetrating tumor [6]. Indeed, these antibody mimicking small size peptides (< 3 kDa) are less toxic and have faster body clearance pharmacokinetics, thus they are potentially more beneficial for specific delivery of the anti cancer drugs. Synthesis and modification of smaller peptides are much easier as they can be radiolabeled and used as alternative radioprobes for tissue targeted imaging [7]. Peptides are also well suited for targeted imaging because of their specific accumulation in desired tissues, which also amplifies their correlated imaging signals for image-guided diagnosis [8]. A group of peptides that has capacity of translocation across membrane were classified as Cell penetrating peptides (CPPs) [9]. CPPs are ideally 4–30 amino acids long and have unique ability to cross plasma membranes through energy independent direct penetration or energy dependent endocytosis mechanisms [10].

CPPs are the most exploited cargos for efficient intracellular delivery of nucleic acids [11], proteins [12], imaging reagents [13], anti-cancer agents as well as small molecules [14] as shown in Graphical Abstract. CPPs are used to translocate various types of cargo molecules across cell membrane; thus, act as transporter. CPPs has dramatically increased in the last decade not only because of reduced cytotoxicity but also because proteins, imaging reagents, and drugs specifically anti-cancer drugs can be linked with these peptides and cross plasma membrane in receptor independent manner. However, various uptake mechanisms associated with CPP are also subject to the length, concentration, physicochemical properties, and charge of the cargo molecules [15]. Tumor cells have certain types of receptors that are up-regulated in comparison to normal cells. These receptors can be exploited to deliver drugs to tumor cells with the help of these CPPs. These peptides have been successfully used to transport various types of drugs, liposomes and nanoparticles for imaging and cancer therapeutics. Despite the fact that CPP-based clinical trials have been dramatically expanded (or are currently underway), no peptide or peptide conjugated with drug has received approval from US Food and Drug Administration (FDA) for cancer therapeutic.

In this review, we will emphasize the benefits and challenges of using theranostic (therapeutic and diagnostic) CPPs for tumor detection and treatment, by focusing more on clinical translation criteria. We will discuss the importance of CPP design and selection criteria based on specific microenvironment characteristics of their targeted tumors. Addressing these factors will enable more efficient drug delivery to tumor tissues, followed by internalization of these peptides into their individual cancer cells. Most common CPPs such as MPG peptides, Pep peptides, and TAT peptides will be addressed in detail. We will also review recent diagnostic and therapeutic approaches, designed based on interaction of peptides with different types of highly-expressed cancer-specific proteases (e.g. urokinase plasminogen activator and matrix metalloproteinases). Finally, we will provide various examples for effective use of CPPs for diagnostic applications such as cancer imaging or development of diagnostic assays, considering the recent clinical trials.

## CELL PENETRATING PEPTIDE AND MECHANISMS OF MEMBRANE TRANSLOCATION

CPPs are short sequence of 4–30 peptides that assist cellular uptake of various cargoes ranging from nanoparticles size to large fragments of nucleotide. Among several criteria that have been proposed over different time, CPPs can be classified as cationic, amphipathic, and hydrophobic peptides on basis of their physical–chemical properties. The cationic peptides have positive net charges and they mainly have arginines and lysines strands while amphipathic CPPs contain hydrophibic polar amino acid such as lysine and arginine as well as hydrophobic nonpolar types of amino acids such as leucine, isoleucine, alanine and valine. On the other hand, hydrophobic CPPs mainly comprise hydrophobic nonpolar residues, due to which they play an important role in cellular internalization.

**Figure 1:**
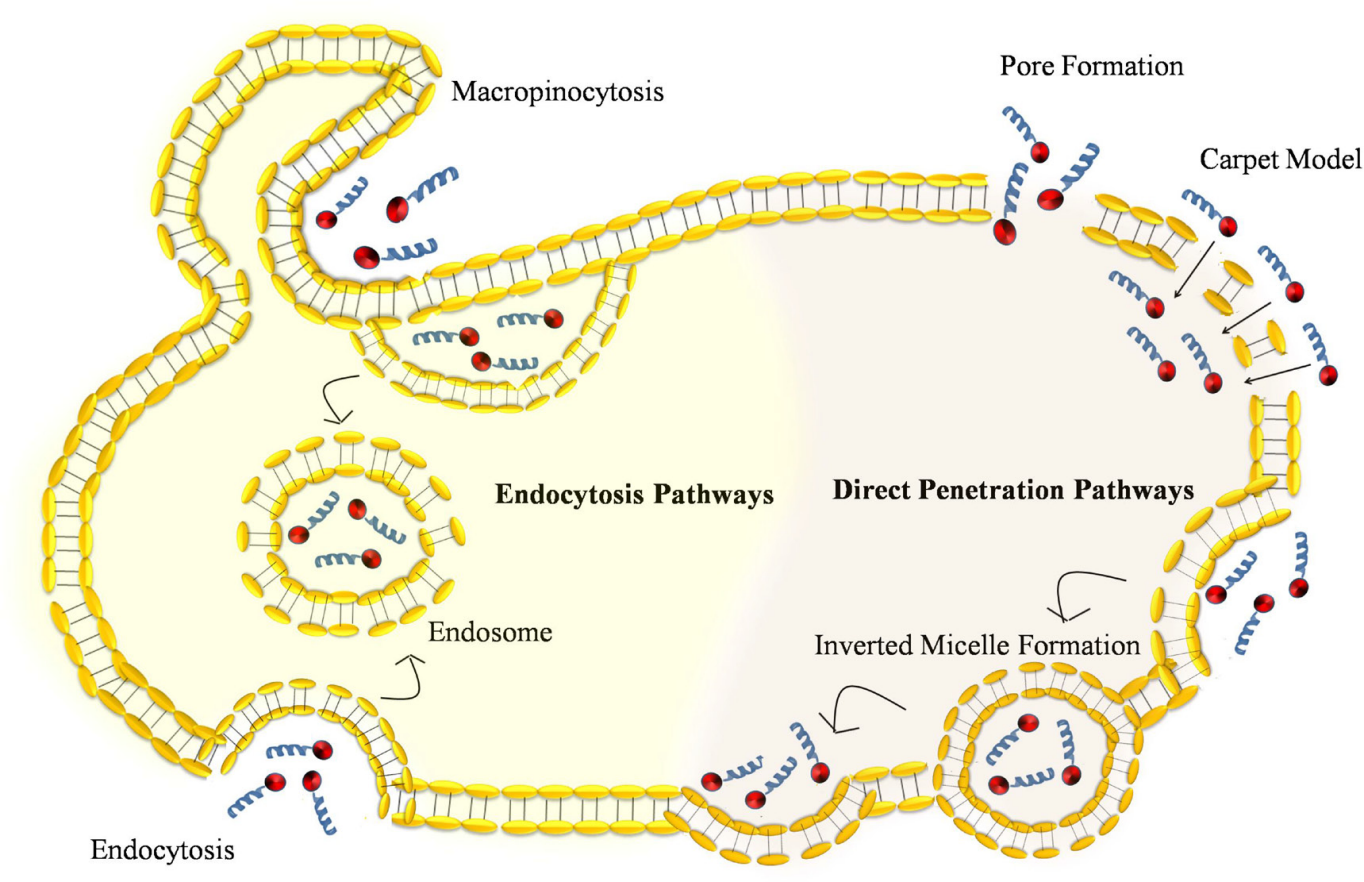
Comparison of various types of pathways used by cell penetrating peptide to facilitate cellular internalization: Direct penetration of CPP-peptide complex into the plasma membrane is an energy independent models such as pore formation, carpet model, and inverted micelle formation Uptake of CPP-peptide complex by endocytosis pathway is an energy dependent process that involves endocytosis and macropinocytosis.

Various physiochemical properties such as types, concentration, size of CPPs or CPP conjugated cargoes play an important role in influencing their cellular uptake. Presently CPP mediated cargo delivery is hampered by lack of their cell specificity and mode of their delivery is not well understood. CPP can use two different routes to enter the cell named as energy-independent direct penetration of the plasma membrane and energy dependent endocytosis mechanism, which are summarize in Figure 1.

### Direct penetration

Energy independent direct penetration happens at low temperatures which involve multiple entry routes that are initially based on the internalization of CPP in the cell through pore formation and membrane destabilization by three different type of models named as barrel-stave pore model, carpet-like model and toroidal pore model [16, 17]. In the barrel stave pore model, hydrophilic peptides are angled parallel to the cell membrane surface. Once there are more than three peptides and pH is high, hydrophilic peptides re-oriented perpendicular from parallel at outer membrane surface to allow the entry of peptides. Since cytosolic pH is lower in comparison to extracellular pH, thus allows to form transient pores [18]. The carpet-like model depicts the interaction between phospholipids in the outer layer of the membrane and positive charge arginines and lysines rich CPPs. Like barrel stave pore model, initially hydrophilic peptides are angled parallel to the cell membrane surface but increased concentration of CPPs rotate and redirect them towards lipid bilayer and then form micelles and pores in it. The toroidal pore is a two-step model in which there is a transition of peptide from inactive form to active form on basis of concentration of peptide. At low concentration, CPP in active form is oriented parallel to plasma membrane surface. Increase in concentration allow the CPP to change from inactive form to active form, thus from parallel to perpendicular orientation to the bilayer and penetrate the hydrophobic regions by irreversible membrane destabilization and release of CPP into cytosolic compartment.

### Endocytosis

Although energy independent direct penetration was initially first anticipated as the principal route to enter the cell but further evidences suggest that energy dependent endocytosis is the main route of entry for internalization for many CPPs [16]. Internalization of CPP by endocytosis includes various models such as macropinocytosis [19], clathrin-mediated endocytosis [20] or caveolin-mediated endocytosis [21]. Which of these pathways will play a role at particular time depends mostly on the basis of how big the cargo molecule is and what is its physiochemical properties. In order for CPP to reach at target site and avoid degradation from the lysosomes present in endosomes, CPP must escape from the endosome to cytosol [22]. Various hypotheses have been proposed for endosomal escape. In one approach pH sensitive domains were introduce in the peptide sequence to disrupt lipid membrane at low pH to felicitate the CPP escape from vesicles [19]. In another approach, histidine residues were introduced into CPPs that increase osmotic pressure in the endosomal vesicle due to proton sponge effect and eventually allow the endosomal membrane to rupture [23]. Another group has used PepFect (PF) peptide alteration by N-terminal stearylation to promote the endosomal escape [24]. The presence of lysosomotropic agent chloroquine CQ equivalent was also shown to be important for improved endosomal escape [25]. Comparison of direct penetration and endocytic pathways has been done in Figure 1. We have also summarized cancer specific CPP, its characteristics, and applications in this Table 1.

**Table 1:** Cancer cell penetrating and targeting peptides, with specific amino acid sequences, characteristics, and applications

Type of peptides	Sequence of peptides	Characteristic features	Applications	References
***Cell Penetrating Peptides***				
**MPG**	GALFLGFLGAAGSTM GAWSQPKKKRKV (27 amino acid peptide)	Amphiphillic Lysine rich domain obtained from nuclear localization sequence (NLS) Follows non-endocytic pathway for delivery.	Delivery of DNA, siRNA, plasmid DNA and Oligonucleotides	[26]
Pep-1	KETWWETWWTEWS QPKKKRKV (21 amino acid peptide)	Similar to MPG and efficiently delivers wide range of peptides and proteins Chemical covalent denaturation or coupling is not required.	Peptides, proteins and PNA analogues delivered using Pep-1	[27]
Pep-2	KETWFETWFTEWSQP KKKRKV (21 amino acid peptide)	Amphipathic peptide Possesses higher stability and potency than pep1	Delivery of nucleic acid and peptide	[27]
Pep-3	KETWFETWFTEWSQP KKKRKV (21 amino acid peptide)	Used to formnanosize complexes Improved cellular uptake	Delivery of nucleic acid and peptide	[28]
CADY	Ac-GLWRALWRLLRSLW RLLWRA-Cya	Secondary amphiphillic peptide and is based on the PPTG1	Delivery of siRNA	[27]
Rath	TPWWRLWTKWHHK RRDLPRKPE	β structure oligonucleotide with a small α helix.	Binds to Plasmid DNA, antibody and proteins	[29]
***Peptides as targeting ligands***				
**LyP-1**	CGNKRTRGC	Nanosystem containing “activators” and “targeted nanoparticle”	Tumor hypoxia and tumor-induced lymphangiogenesis	[30]
SP5-52	SVSVGMKPSPRP	Conjugates specifically to DSPE-PEG liposomes	Inhibits angiogenesis	[31]

## CPPS AS DRUG CARRIERS

Crossing the plasma membrane barrier is one of the main obstacles against intracellular drug delivery, especially for charged protein molecules with larger sizes [32]. CPPs can be modified feasibly for efficient translocation through cell membrane. For example, amino acids 43–58 were considered as the transcription factor homeodomain of third helix of *Drosophila melanogaster* antennapedia. Hence, the first CPP peptide developed was named as antennapedia peptide (Antp) [33]. Transportan (TP) is another peptide that is 12 amino acids long, obtained from galanin, a neuropeptide. Linking of TP with mastoparan (14 amino acids long wasp venom derived peptide), results in the formation of a chimeric peptide [34]. There are also various synthetic peptides, such as polyarginine, that have been designed for this purpose [35]. The major applications of chimeric and synthetic peptides are in intracellular delivery of drugs via efficient transcytosis compared with other types of peptides. The exact mechanism for peptide uptake via plasma membrane is still unclear but the primary assumptions are based on electrostatic interactions.

Complexes formed by conjugation of drugs and peptides can also be exploited for targeted drug delivery. It was shown that efficient targeting of peptide-drug complexes are mainly achieved via endocytosis, but there are also several other factors responsible for their uptake such as concentration of drug and peptide, cell surface, and lipid components of plasma membrane [36]. CPP-based targeted drug delivery is possible by covalent bonding between peptide and drug molecules to form a stable complex. The covalent bonding of CPP-peptide can be accomplished by cloning or conjugation chemistry via cross linking of fused products (CPP-peptide complex) [37]. The elaborated procedure for conjugation of cell penetrating peptide has been thoroughly described [38]. Cell penetrating peptide-mediated delivery of bioactive molecules can be achieved by pinocytosis followed by endosomal escape [39]. Individual cell penetrating peptides or their conjugates with small molecules can be internalized inside the cells via Van der Waals interaction and hydrogen bonding interaction. These non-covalent interactions include both hydrophilic and hydrophobic amino acids residues [40]. The hydrophilic amino acids help in targeting by increasing the water solubility, while the hydrophobic residues help in formation of complexes by different tethering mechanisms. In recent review, various types of CPPs, internalization mechanism and conjugation strategy together with potential application in cancer therapy are highlighted [16]. The role of MPG, Pep, and, TAT peptides and their advantages are described below.

### MPG peptides

**Figure 2:**
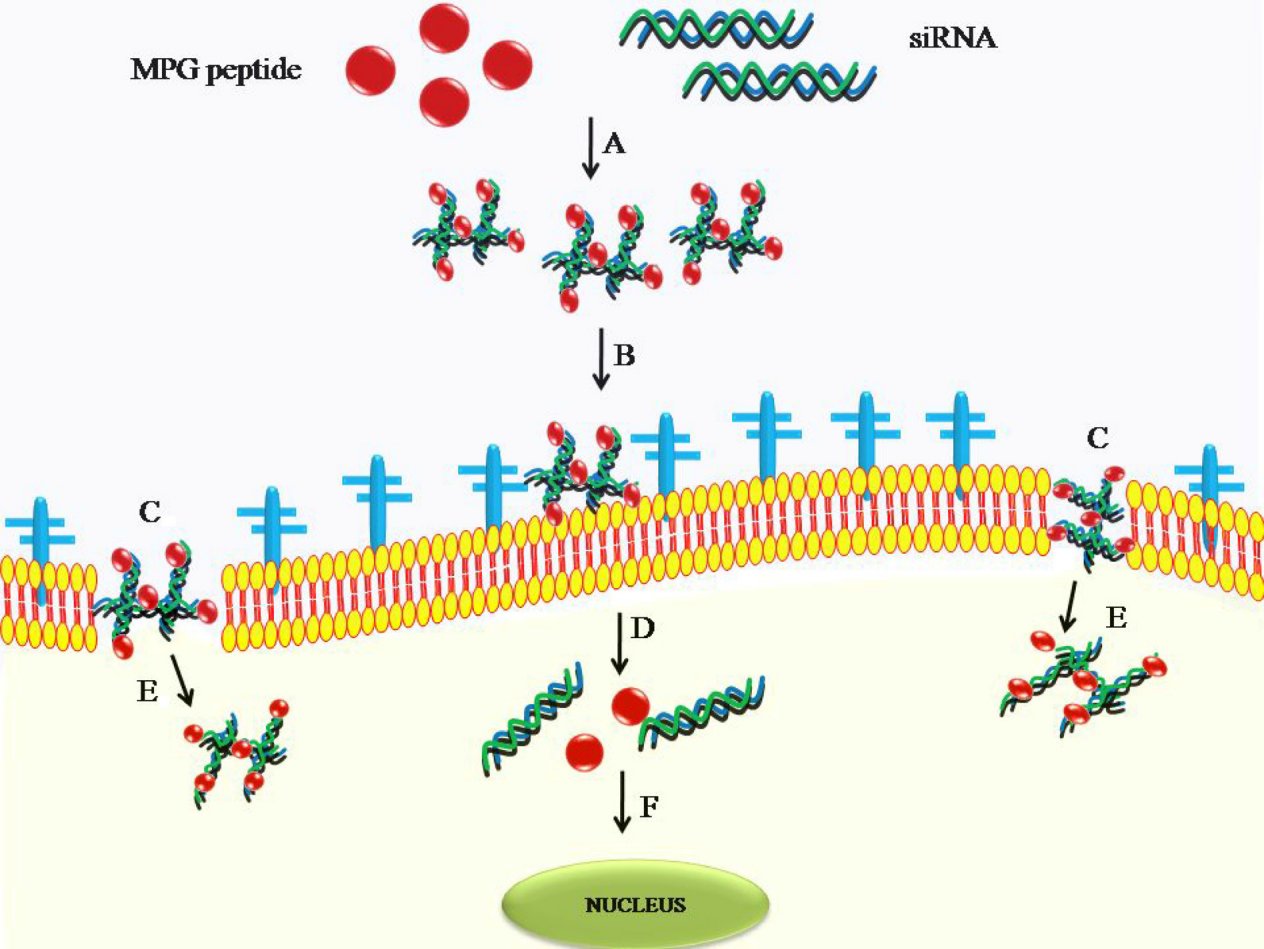
Delivery of siRNA using cell penetrating peptide as a cargo (**A**) MPG-siRNA forms a complex through electrostatic or hydrophobic interactions followed by (**B**) interaction of the complex with the cell surface proteoglycans, or (**C**) direct penetration of the of the complex through the lipid phase of the cell, (**D**) and the complex is released inside the cytoplasm (**E**) which is followed by dissociation of the complex inside the cytoplasm by action of proteases and (**F**) and is finally targeted towards the nucleus.

MPG peptides are amphiphilic peptides consist of three domains: hydrophobic, lysine rich, and a linker. These three domains provide efficient targeting, uptake, interaction with nuclear material, and determine flexibility and integrity of the peptide molecules [41]. These characteristics also make peptides as perfect choices for delivery of oligonucleotides and plasmid DNA into a variety of non-adherent and adherent cells [42]. Functionality of many non-viral vectors dependson the activity stage of cell cycles, which sometimes poses hurdles in their effective delivery. However, performance of MPG peptides does not depend on the cell cycle progression for nuclear envelope breakdown. Very high siRNA delivery efficiency up to 90% and rapid translocation of siRNA inside the nucleus has been reported when MPG is used as transfection vector [42]. Cell entry for MPG–siRNA is independent of the endosomal pathway. Instead, the cellular uptake of MPG–siRNA is associated with MPG peptide functionality to interact with the cell membrane lipids, thus leading to the formation of temporary trans-membrane alpha helical or beta structures. These temporary structures alter cell membrane organization and thus allow insertion of the complex into membrane, usually followed by translocation due to membrane potential (Figure 2) [43].

### Pep- peptides

Pep peptides are amphipathic in nature and form non-covalent complexes with their selected cargo to facilitate peptides and proteins delivery inside the cell. They help to overcome the drawback of drugs bioavailability as indicated by *in-vivo* studies. For example, Pep-1 peptide is quite similar to MPG. The hydrophobic domain in Pep-1 fosters the internalization of small molecules and large proteins [44]. Numerous alterations have been suggested for Pep-1 to improve the stability and potency. Pep-2 is a modified cargo of Pep-1 that specifically differs at two positions in hydrophobic domain and helps in the formation of stable carrier for efficient uptake. The presence of aromatic residues in Pep-1 favors the deformation of lipid bilayer on cell membranes, a mechanism that was used to design Pep-3 with enhanced uptake up to 92% [45]. Other chimeric peptides in this series are CADY with carrier ppTG1 for high efficiency siRNA delivery at low drug concentrations [46] and Rath for plasmid oligonucleotide, IgG and GFP delivery [29].

### TAT peptides

TAT (transactivator of transcription) is derived from HIV and is a CPP peptide. It was first fused with β –galactosidase and used for delivery applications throughout brain tissue [47]. Various reports have suggested the importance of TAT fusion for penetration of oligonucleotides, peptides and proteins [48]. Conjugation of p53 gene with TAT peptides led to the activation of p53 gene with successful targeting of human cancer (TA3/St and Namalwa lymphoma tumor). Met peptide in conjugation with TAT showed inhibition of hepatocyte growth factor (HGF) in liver [49]. CT26 mouse colon adenocarcinoma cells targeted with TAT and fused with chitosan or doxorubicin, showed two-fold higher inhibition as compared to controls. Tumor targeting functionality can be further enhanced by designing pH sensitive TAT-PEG complexes with capabilities to release drugs during their penetration into the cell membranes, due to acidic pH of the tumor cells [50].

Overall, CPP mediated delivery of vehicle has significant advantages for treatment of a wide range of diseases including cancers. As discussed in this review, CPPs can be used for targeted delivery and release of various drugs and therapeutic agents. The drug delivery efficiency depends on proper design of the linker, nature of the CPPs (hydrophobic or hydrophilic domains), and specific characteristics of the carrier-CPP complexes for efficient cell membrane penetration, endosomal escape, and intracellular trafficking. Characterization of these specific parameters is crucial for designing efficient and potent CPPs.

## CPPS AS LIGANDS FOR TUMOR TARGETING

**Figure 3:**
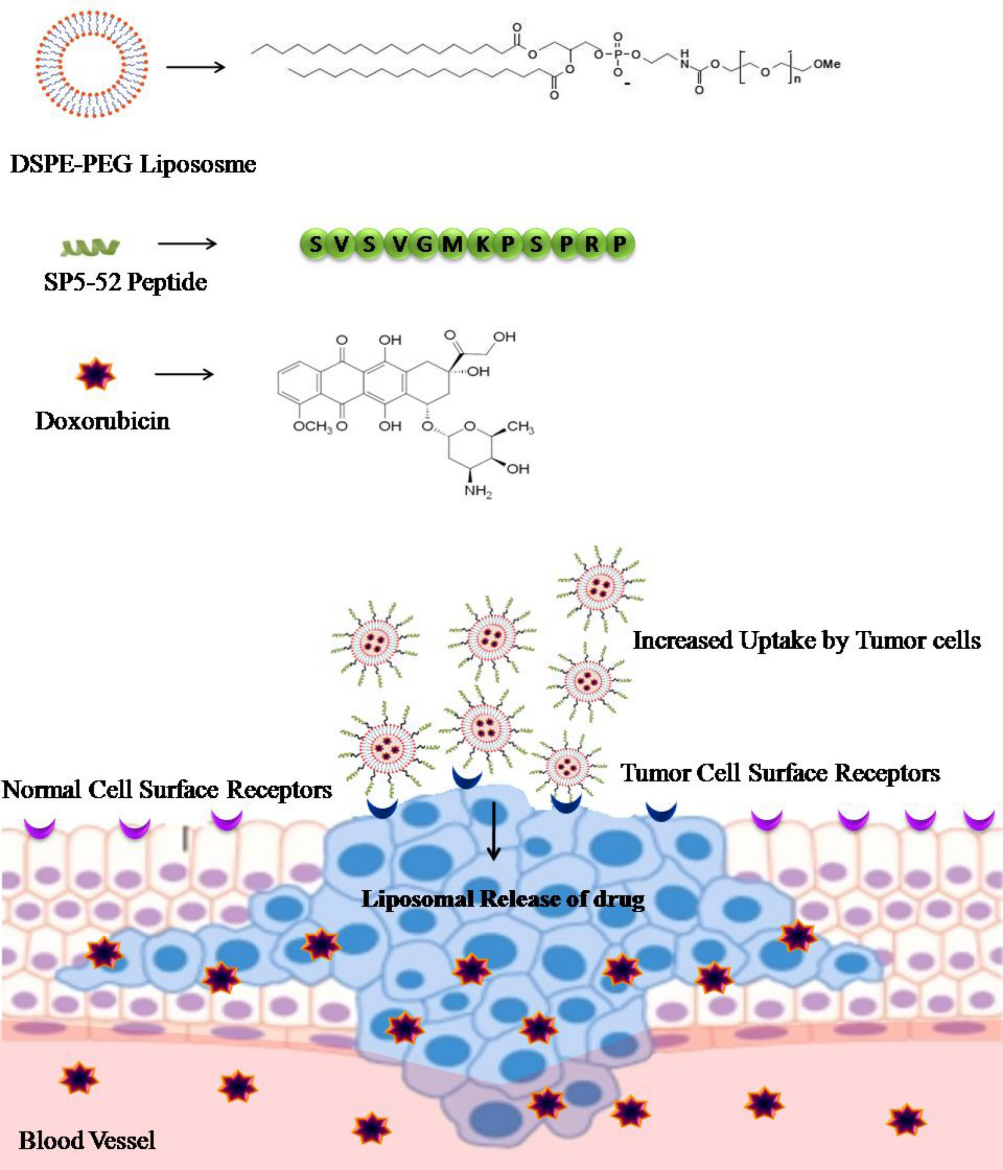
Delivery of doxorubicin using DSPE-PEG liposome as a carrier: SP5-52 peptide is conjugated onto the surface of doxorubicin loaded DSPE-PEG liposomes SP5-52 contains consensus sequence that allows selective targeting of tumor cells and increased rate of delivery of doxorubicin drug to the tumor cells as compared to control/normal cells.

Ligands that are currently used as specific targeting agents are proteins, peptides, carbohydrates, vitamins, antibodies and aptamers. The selection of specific peptides can be strategized via chemical and biological approaches [51] for different targeting studies related to pancreatic β cells [52], malignant cells [53] and integrin [54]. Moreover, labeled peptides, self-assembled peptides and aptameric peptides have also widened the scope of tumor targeted imaging with less toxicity [55]. Wu *et al*. [31] showed targeting of tumor blood vessels by SP5-52 peptide conjugated with PEGylated distearoyl phosphatidyl ethanolamine (DSPE-PEG) liposome. The SP5-52 peptide contains consensus sequence that differentiates tumor blood vessel from a normal blood vessel and showed more than eight-fold increase in accumulation of SP5-52 peptide compared to control cells. Further, loading of doxorubicin into SP5-52-DSPE-PEG liposome particles, which decreased the formation of tumor blood vessels, which resulted in longer survival of cancer in xenograft mice. The study showed that SP5-52 peptide can be efficiently used for targeted delivery of drugs to solid tumors (Figure 3).

LyP-1 is also another targeting ligand that has shown cytotoxic activity resulting in inhibited metastasis (for example in MDA-MB-435 cells) and decreased lymphatic vessel. LyP-1 peptide was loaded on gold nanorods and nanoparticles for targeting MDA-MB-435 tumor cells. Accumulation of gold nanorods at the site of tumor generated photothermal effects after laser irradiation. LyP-1, when loaded on gold nanoparticles alone or when loaded with doxorubicin, helped in binding with p32 protein (a mitochondrial protein that has both increased expression and aberrant localization on tumor cell membranes). A noticeable decrease in tumor size and volume was detected in this investigation [30].

Additionally, a chimeric peptide named as rabies virus glycoprotein (RVG) was also used for *in-vivo* siRNA delivery to brain [56]. In this study, RVG peptide was linked with green fluorescent protein (GFP) labeled siRNA. Expression of GFP increased only in the brain, without significant uptake in other tissues, thus indicating specific and efficient delivery. Similar results were also reported by using other peptides such as RVG-9R complexes with siRNA that can cross blood brain barrier and target japanese encephalitis virus [57].

## CPPS FOR TUMOR DRUG DELIVERY

Overexpression of proteases is related with wide range of diseases including cancer. Proteases are cellular enzymes that act as biomarkers in regulatory pathways and are involved in amide bond cleavage between adjacent amino acids. These proteases could become a potential tool, if used as drug delivery or imaging agents to target cancer cells [58]. Extensive studies have been done on matrix metalloprotienases (MMPs), and urokinases [59, 60].

MMPs are zinc dependent endoproteases, and play an important role in the extracellular matrix proteins degradation, usually leading to cancer invasion or metastasis [61]. Overexpression of these MMPs (MMP-2, 9, 11, 1, 3, 13, 11, 14 and 7) in malignancies has been correlated in different tumors (*e.g.* gastric, breast, colon, lung, and esophageal). The mechanism that underlies the therapeutic effects of MMPs involves higher permeability and higher retention. In a recent study, apoly (ethylene glycol) diacrylate hydrogel wafer was incorporated with an activable MMP protease (MMP-2 and MMP-9). When human U-87MG brain tumor cells were grown to full confluency in conditioned media, overexpression of MMP-2 and 9 occurred that promoted the release of cisplatin from the wafer [62].

In another study, dextran-peptide-methotrexate conjugates were used for targeted drug delivery to HT-1080 tumors in mice. Conjugates with MMP sensitive peptide linkers were compared with conjugates having MMP insensitive linkers. The pro-drug was cleaved by MMP-2 substrate polymer when used in conjugation with methotrexate that inhibited tumor growth [63]. For instance, the amino (NH_2_) group of dioleoyl phosphatidyl ethanolamine (*i.e.*DOPE) was linked to PEG-conjugated MMP-2 substrate molecules. This PEG-peptide-drug (PEG-PD) complex was contained inside galactosylated liposomes (Gal-PEG-PD-liposome). Higher concentration of MMP2 showed increased uptake of Gal-PEG-PD-liposomes by HepG2 cells, resulting in PEG-PD cleaving by MMP2 [64].

Urokinase plasminogen activator (*i.e.* uPA) is another cancer-associated protease (CPA) that is known to be highly up-regulated in angiogenesis and used as an indicator of invasion and metastasis [65]. uPA responsive hydrogel, named as KLD-12 peptide was described to control the cleavage of r7-KLAcytotoxic peptide that inhibits extracellular matrix (ECM) degradation [66]. In another study, Basel *et al*. [67] designed protease anchored liposomes contained docetaxel (DTX) to release drug based on changes in their osmolarity. Presence of uPA caused initial polymer degradation, followed by changes in osmolarity and swelling of the liposomes, thus resulting in triggered release of the drug. This study showed that such protease-sensitive liposomes can be effectively used for more specific targeting of tumors.

Overall, over-expression of proteases can be seen in association with the onset of various diseases and works on the principle of proteolytic activity. Thus, proteases can also be linked with an imaging probe and used in the early stage diagnosis of diseases related to different organs that will be discussed in Section 6.

## CPPS IN CANCER DIAGNOSTICS

Recent advances in molecular imaging tools have produced numerous approaches for designing smart probes such as radio-labeled small molecules, monoclonal antibodies, and antibody fragments for imaging and diagnostic procedures. Although some achievement has been accomplished, the usages of these probes were not clinically effective, primarily due to their low specificity and inadequate target permeability. Peptides have been progressively used as imaging probes, due to high binding affinity, specific uptake, high stability *in-vivo*, rapid clearance from non-specific target, and retention in the target. Recently, selected number of CPPs has been exploited to target a range of biomarkers and disease-linked receptors. Various CPPs have been used to deliver radioisotopes as diagnostic agents. RGD peptide conjugated 18F radiolabel agent was used to target integrin expressing tumors [68]. Similarly, Cyclic-RGD peptide conjugated with [99mTc(HYNIC-tetramer)(tricine) (TPPTS)] radiolabel agent was used to target integrin-positive MDA-MB-435 breast cancer cells [69]. These targeting CPPs may be conjugated to optical imaging moieties (such as fluorophore-labeled or activable probes), nanoparticles, polymers, and contrast agents [70]. Activable optical probes are peptide-based molecules that have fluorescently quenched fluorophores, cleavable peptide linker, and a quencher attached at the opposite ends of the linker [71]. Cleavage can happen due to presence of a protease specific recognition site, a phenomenon called proteolytic cleavage. Such cleavage results in increased fluorescence intensity, while quenching of the primary substrate does not provide any signal at its native state. A flow cytometry-based assay was established for detection of separase enzyme activity in human histiocytic lymphoma U937 cells [72].

**Figure 4:**
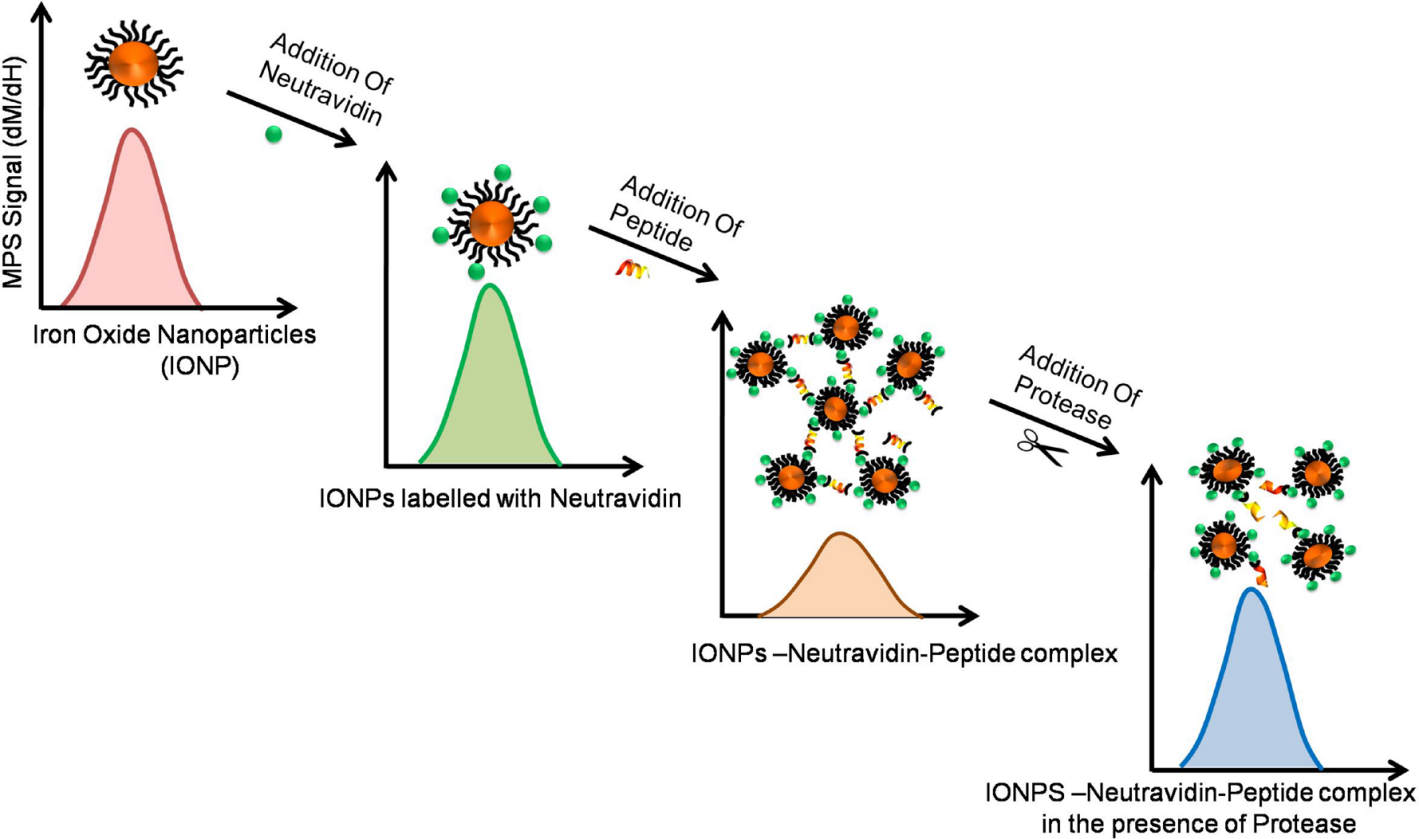
Role of CPPs in cancer diagnosis: Changes in magnetic properties and magnetic relaxation of iron oxide nanoparticles conjugated with cleavable CPPs may be effectively used for detection of proteases expressed by cancer cells This sensitive assay can be used for detection of proteases by using specifically designed activable nanosensors.

Substrates can also be designed to generate other types of signals (such as magnetic) after their proteolytic cleavage [73]. For example, we developed activable nanosensors with iron oxide nanoparticles (IONPs), for detection of proteases secreted by pancreatic and fibrosarcoma cells. IONPs were labeled with neutravidin and CPPs were labeled with biotin at both ends with a protease specific recognition site in the middle [74]. When IONPs and CPPs were mixed, aggregation of IONPs occurred due to neutravidin-biotin interaction. Addition of supernatant from cells expressing proteases (such as MMP-2 or trypsin) to the aggregated complex resulted in the cleavage of CPPs and re-dispersion of IONPs-N-P complex, causing well-defined changes in their magnetization (*i.e.* magnetization rate and saturation magnetization) [75]. Here, the full-width at half maximum and peak height of magnetization rate of the nanoparticles (dm/dH) represents the changes in magnetic response of the nanoparticles in a magnetic particle spectrometer (MPS) system (Figure 4).

In a separate study, the existence of PEG-MMP-2 and MMP-7 substrates, which are biomarkers for various types of cancers, including but not limited to pancreatic, hepatic and, breast, restricted self-assembly of magnetic nanoparticles and caused decreased T2 relaxation in magnetic resonance imaging (MRI). Fluorosceinisothiocyanate (FITC)-labeled polyarginine cell internalizing peptide, and TAMRA labeled protease-cleavable polyethylene glycol (PEG) were prepared by linking the amine terminus of an MMP-2 cleavable CPP peptide substrate, NH2-GK(TAMRA)GPLGVRGC, to NHS-PEG (M.W. 10 kDa). This conjugate was linked to nanoparticles and used for *in-vivo* studies. Results showed efficient uptake by HT-1080 human fibrosarcoma cells, as PEG prolongs the blood circulation and facilitates the cleavage of MMP-2 by substrate, due to over-expression of MMP-2 in tumor. MRI and fluorescent imaging showed enhanced fluorescence intensity in treated tumor as compared to control, proved the role of MMPs in catalytic activity [76].

In a recent study, dendrimer nanoparticles were labeled with activable nanosensors for fluorescence or MRI-based imaging [77]. The probes were designed with MMP substrate CPP carrying Cy5.5, PEG and a quencher at N- and C- terminals, respectively. *In-vivo* studies showed increased fluorescence intensity and reduced activation time in SCC-7 tumor (squamous cell carcinoma) bearing mice. A separate study showed that this nanosensor activation is not necessarily limited to a specific tumor. Duijnhoven *et al*. [78] reported similar results in MMP2/9 tumor bearing mice as well as control mice, indicating that the activation is most likely caused by cell membrane bound enzymes present in the vasculature rather than specifically by the tumor. In recent review, herb based drugs delivery on specific tumor site was achieved with CPP and tumor-targeting peptide-modified nanocarriers [79]. Further investigations are required to unravel the exact mechanisms involved in inactivation of such nanosensors by various proteases at different stages of diseases. Advanced nanosensors can be designed for pro-drug development, discovering cellular signaling pathways, and generating multimodal imaging signals. In addition, proteases can be used as biomarkers by virtue of their catalytic activity and to further activate other matrix metalloproteinases upon cleavage. Nanoparticles labeled with these proteases can be used for developing diagnostic assays, cancer therapeutics and imaging, depending on detection limits of the technique used. Several CPPs have delivered various types of contrast agents, which could also be used for diagnosis of variety of tumors [80]. For example, RGD peptide was exploited to deliver Gd-DOTA contrast agent to target avb3 receptors in H-ras12V transgenic mice [81].

To date, many of radiolabeled peptides have been clinically used for diagnosis which are recently reviewed here [82]. CPPs have been chemically engineered with the aim for enhanced metabolic stabilities and more favorable pharmacokinetics. Radiolabeled RGD peptide which have been used in the clinical trials including ^99m^Tc-αP2, [^18^F]Galacto-RGD, [^18^F]Fluciclatide, [^18^F]RGD-K5, ^18^F-FPPRGD2, [^18^F]Alfatide, [^68^Ga]NOTA-PRGD2, and ^99m^Tc-3PRGD2 [81]. Chemical modifications, such as glucolysation, PEGylation and multimerization, have been used to improve the imaging quality. [^18^F]Galacto-RGD, was the first RGD based PET tracer that was tried in human showing specific binding and rapid renal clearance in cancer patients. Phase I clinical trial of [^18^F]Fluciclatide with tumors was visible on the PET images in breast cancer patients [83]. [^18^F]FPPRGD2 was first dimeric RGD peptide approved by FDA that was used for human trial demonstrating positive results in glioblastomamultiforme (GBM) [84].

## PEPTIDES IN CANCER THERAPY: LESSONS FROM PRECLINICAL AND CLINICAL TRIALS

Several preclinical assessments with CPP-conjugated imaging molecules and drugs have provided encouraging results for cancer imaging and therapy. CPPs have also been used in several pre-clinical trialfor the treatment of oncological diseases [85]. Major emphasis was given to p53, a tumor suppressor gene. Tumor progression depends on the loss of p53 function due to mutations of the gene, which are present in various types of human cancers [86]. Therefore, to reestablish the endogenous level of p53 in tumor cells, a transducible and proteolytically stable peptide [751TD$DIF], named RI-TAT–p53C0 was developed. This compound comprises a retro-inversoD-isomer peptide derived from the C-terminal regulatory domain of p53 linked to TAT_47–57_ [87]. Wild-type p53 was induced by C-terminus of p53 to activate apoptosis and this restore the transcriptional trans-activating function of mutant p53 proteins. Systemic delivery of the RI-TAT–p53C peptide in preclinical lymphoma models caused in substantial increases in lifetime and the production of disease-free animals.

DTS-108, water-soluble compound containing chemotherapeutic agent SN38 are shown to have anti-tumor affect in colon, lungs and breast cancer [88]. Amphipathic peptide MPG-8, another CPP has been used to form nanoparticles with siRNA for efficient delivery and target cyclin B1 in mice. Surface of MPG-8/siRNA particles were functionalized with a cholesterol moiety and later injected in xenografted tumors model to significantly reduce the tumor size [89]. Alternatively, siRNA was also delivered for therapeutic application in cancer model. Double-stranded RNA-binding domain was linked with TAT fusion protein to binds siRNAs and to use it as vehicle for delivery of EGFR and AKT2 [90]. These pre-clinical results have prompted clinical trials in some cases. Table 2 contains a list of various CPPs and their potential applications in both preclinical and human clinical trials for different cancer diagnosis and treatment purposes.

**Table 2:** List of various CPPs designed for preclinical and clinical cancer diagnosis and treatment

CPP	CPP-CARGO	APPLICATION	REFERENCE
RI-Tat-9	RI-TAT–p53C	Peptide-cargo used for treatment of peritoneal carcinomatosis or lymphoma in mice	[89]
TAT	TAT-DRBD/siRNA	It causes tumor specific apoptosis and aids in treatment of intracranial glioblastoma cancer in mice model	[88]
MPG	MPG-8/siRNA	MPG-mediated targeted siRNA delivery to suppress tumor growth in mice with tumor xenografts.	[87]
BR2	BR2-scFv	Delivery of BR2-scFv fusion protein inhibits cancer cells proliferation	[90]
p28	p28-p53	Induces p53 mediated apoptosis; being used in clinical trials in humans after success in preclinical stage	[91]
p28	p28-p53	Evaluation of safety, toxicity and dosage in children with recurrent CNS malignancies	[92]

Azurin-derived CPP was recently used in a clinical trial (phase I) to treat refractory tumors. p28 is a 28-amino-acid peptide, which once penetrated in nucleus of cancer cell bind with tumor suppressor protein P53 and inhibits p53 ubiquitination that can reduce CDK2 and cyclin A1 level and stop tumor growth in the G2/M cell cycle stage, by cell apoptosis due to inhibiting proteasomal degradation. These Phase I clinical trials focused on safety, pharmacokinetics, maximum tolerance dose and efficacy of p28 in patients with p53^+^ solid tumors which are resistance to conventional method of treatments and therapies. p28 also shows antitumor activity and minimal toxicity with no immunogenicity and was well tolerated. Importantly, this was also highly successful among those patients having recalcitrant disease who had earlier rejected prior treatments. Significant improvement in the survival of p53-positive advanced solid tumors was observed after treatment for thrice per week for total duration of four weeks. The outcomes of this clinical trial strongly indicate that CPPs can be used clinically in cancer therapy [17, 91]. Additionally, conjugation of H.8 to Azurin can cross blood brain barrier and act selectively on glioblastoma multiforme, without showing any non-specific cytotoxicity in phase-I clinical trial [92].

Some latest findings have been focused on dissecting the role of p28 in treating young patients with repeated or progressive high grade glioma. In these phase-I clinical trial studies, p28 was injected 3x/week for 4 weeks and repeated every 6 weeks in pediatric patients with refractory glioma, to assess the toxicity and best dose of p28. This phase established that p28 is quite satisfactory tolerated in children with repeated high-grade glioma at the adult suggested phase II dose [92]. Another study suggests that p28 has an anti-angiogenic effect by inhibition of VEGFR-2 kinase activity. The study reported that p28 reduced the phosphorylation of FAK and Akt, suggesting that p28 stimulates a pFAK-mediated loss of migration and motility, in addition to p28 induces Akt-associated survival. This proof-of-concept Phase II clinical trials demonstrates that p28 enhances the cell cycle inhibition and reduces tumor cell proliferation [93]. To reduce the side effect and attain higher beneficial levels in rectal cancer patients, CPP was conjugated to SN38. DTS-108 is a soluble pro-drug of SN38, which releases SN38 from CPP by cleavage of esterase bond. This Phase I clinical trial discussed tolerated dose, dose-limiting toxicities and pharmacokinetics after DTS-108 injection in patients with highly metastatic cancers [94].

CPPs have also been used in clinical trial as a transporter for cancer specific drug carriers. For instance, BR2 (a 17-amino acid long cell penetrating peptide) was fused with scfv and targeted towards K-ras mutated HCT116 cells. Br2-scFv complex was shown to cause increase rate of apoptosis in K-ras mutated cells without causing toxicity to normal cells. These outcomes indicate that BR2 has a huge potential to be used as a cancer specific drug carrier in clinics [95]. Indeed data obtained from preclinical and clinical trials have validated the use of CPPs as a vehicle for therapeutic molecules, thus permitting them to reach their targets. Overall, the accelerated rate of developing various CPPs and their widespread use in preclinical, and clinical trials emphasize their potential impact in cancer diagnosis and treatment in a near future.

## CONCLUDING REMARKS AND FUTURE PERSPECTIVE

CPPs can not only be exploited as therapeutic molecules that can cross cell membrane, but also used as promising tools for delivery of diagnostic as well as therapeutic cargos including genes, nanocarriers, anti-cancer drugs and imaging agents inside cells. Conjugation of these imaging agents such as fluorophores to CPP enables efficient detection of the diseased tissues for diagnosis purposes. Early diagnosis of tumors and improved targeted drug delivery is required to increase the effectiveness of cancer therapy and depends on identification and development of tumor-targeting agents. Use of CPPs forcancer drug delivery is considered as unique and most promising approaches for treating cancers and has profound potential to change the field of cancer theranostics in the near future. Although promising results have been reported for *in-vitro* and *in-vivo* studies, no CPP or CPP-drug conjugate has received FDA approval for cancer therapeutics so far. Therefore, additional studies are required to conclusively address obstacles between preclinical efforts and FDA approval criteria, considering significant potentials of CPP class of peptides in the clinics.

It is crucial to explore novel approaches for delivery of CPP and prodrugs not only to the desired tissue or organ, but also inside the targeted cells, to achieve efficient treatment. Advancements in the processing and design of antagonistic peptides with extraordinary tumor-penetrating properties will play a substantial role in forthcoming cancer treatments. CPP can also be used as vector for intracellular delivery of transcription factor [96]. Therefore, it is foremost important to investigate various approaches for delivery of CPPs not only to the desired tissue or organ, but also inside the targeted intracellular organelles viz. lysosomes, nucleus and mitochondria, to accomplish efficient treatment. Short blood plasma half life is another drawback as delivery carrier may be degraded before reaching the target site due to presence of proteases. Few studies have suggested to increase the stability of CPPs by using D isoform as they are less sensitive to protease degradation than the L enantiomer. Degradation of CPP by acidic pH in endosome or lysosome is another drawback. Thus, CPPs should be designed in such a ways that it should promotes efficient endosomal escape to speedy liberation of carrier from endosome into cytoplasm.

In recent time, several other strategies including modulating the switch between dormant CPP and active CPP is also gaining attention, thus allowing inactivated CPP to reactivation of CPP on basis of change in pH, temperature and enzymes [97]. Targeting of the ECM proteins, growth factors along with their receptors, coagulation factor proteins, thrombin proteins, and serpins with CPPs should also be considered for future preclinical and clinical studies.
